# Current practices and perceptions of ChatGPT in gynecologic oncology: results from a cross-sectional questionnaire (TRSGO-AI-001)

**DOI:** 10.1186/s12909-026-09192-w

**Published:** 2026-04-15

**Authors:** İbrahim Yalçın, Salih Taşkın, Gözde Şahin, Faruk Köse, Fırat Ortaç, Esra Bilir

**Affiliations:** 1 Department of Gynecology and Obstetrics, Dokuz Eylül University, Izmir, Türkiye; 2 Division of Gynecologic Oncology, Department of Obstetrics and Gynecology, Ankara University School of Medicine, Ankara, Türkiye; 3 Department of Gyneacologic Oncology, University of Health Sciences, Cam Sakura City Hospital, Istanbul, Türkiye; 4 Department of Obstetrics and Gynecology, Acıbadem Mehmet Ali Aydinlar University, İstanbul, Türkiye; 5 Department of Gynecology and Obstetrics, University Medical Center Göttingen, Robert-Koch-Straße 40, Göttingen, 37075 Germany; 6 Department of Gynecologic Oncology, Koç University School of Medicine, İstanbul, Türkiye

**Keywords:** Large language models, ChatGPT, OpenAI, Gynecologic oncology, Physician

## Abstract

**Background:**

We aimed to assess current practices, perceptions, and perceived effectiveness of ChatGPT among gynecologic oncology professionals in academic and clinical settings.

**Methods:**

A 23-item, international online survey was conducted between July and September 2025. Gynecologic oncology professionals were recruited via social media and snowball sampling. We evaluated demographics, awareness, utilization, and perceptions of ChatGPT-3.5 across different domains.

**Results:**

Our respondents(*n* = 111) were predominantly aged 36–45 years(55.0%), from Türkiye (90.1%), and affiliated with academic institutions(87.4%). Nearly all respondents(99.1%) were aware of ChatGPT, and 66.7% reported professional use, primarily for literature summarization(52.3%), teaching(42.3%), and academic writing(36.9%). Direct clinical use remained somewhat limited(17.1%), with minimal engagement in patient communication(7.2%). Misinformation was the most commonly reported concern(66.7%). Perceived effectiveness was highest in academic writing, literature summarization, and teaching, whereas patient communication, clinical decision support, and exam preparation were rated somewhat low. Overall, 60.3% indicated that ChatGPT contributed meaningfully to their work, 45.9% supported its integration into gynecologic oncology education, and 58.5% would recommend it to colleagues. Participants < 45 years and those with < 5 years of clinical experience reported significantly more frequent ChatGPT use in their professional activities(p-value = 0.026). Those with greater clinical experience were more likely to use ChatGPT for writing purposes(p-value = 0.039), whereas those < 45 years used it more often for clinical decision-making(p-value = 0.015).

**Conclusions:**

ChatGPT is widely recognized and used for academic and educational tasks in gynecologic oncology, yet its clinical applications remain limited. Future research should optimize large language models for clinical use, evaluate comparative outcomes across diverse models, and investigate their integration into multidisciplinary care.

**Supplementary Information:**

The online version contains supplementary material available at 10.1186/s12909-026-09192-w.

## Introduction

In recent years, large language models(LLM) such as Chat Generative Pre-Trained Transformer(ChatGPT) by OpenAI, Gemini by Google DeepMind, and LLaMA by Meta have increasingly invaded to the daily life, extending their influence into academic and clinical domains. A systematic review of LLMs in healthcare reported that ChatGPT, launched in November 2022, was the most widely used conversational large language models, appearing in 92% of the included studies [1]. The review highlighted the large language models’ promising performance in tasks such as text summarization and providing general medical knowledge to patients, demonstrating relatively high accuracy [1].

Gynecologic oncology (GO) provides a particularly demanding and clinically meaningful setting for evaluating LLMs because it demands more than just data processing; it requires nuanced and multidisciplinary clinical judgment. Managing malignancies like ovarian, cervical, and endometrial cancers necessitates a seamless integration of surgical, systemic, and radiotherapeutic strategies tailored to the individuals. Beyond technical complexity, the field is defined by deeply personal patient interactions highlighting the delicate balances of fertility preservation, survivorship, and palliative care. Because GO bridges the gap between medical and surgical precision and also empathetic communication, it offers a unique opportunity to evaluate how LLMs might practically enhance clinician education, evidence-based medical decisions, and most importantly support the patient experience.

The use of ChatGPT in GO has been reported in several contexts, including designing clinical trials, its feasibility in tumor boards, and assisting in the preparation of scientific abstracts [2–4]. A systematic review of LLMs found that ChatGPT-4 provided correct recommendations in 75% of cases, with variable concordance with established guidelines (70% for the National Comprehensive Cancer Network^®^(NCCN^®^), 60% with the European Society of Gynaecological Oncology(ESGO)) and demonstrated higher recommendation quality for ovarian cancer compared to endometrial cancer [5]. A study assessing ChatGPT’s responses to frequently asked questions about cervical cancer found that the model provided accurate and satisfactory answers; however, caution is advised when consulting it on treatment-related issues [6]. Thus, it is important to remark that LLMs are gaining attention in GO for their potential to support a range of tasks, including the creation of patient-friendly educational materials, summarization of rapidly evolving clinical guidelines, facilitation of shared decision-making, and assistance with literature synthesis and clinical documentation. While LLMs may eventually assume a supportive role in everyday clinical work, the ultimate diagnostic and legal responsibility remain firmly with the physician.

In a prospective comparative study assessing the performance of Chat Generative Pre-Trained Transformer–omni(ChatGPT-4o) in responding to questions related to endometrial cancer, reported that ChatGPT outperformed the oncologist in terms of accuracy(*p*-value < 0.001), empathy(*p*-value < 0.001), and completeness, (*p*-value < 0.001) [7]. Another study highlighted ChatGPT’s tendency to recommend potentially excessive genetic testing within the field of GO [8].

LLMs’ capacity to quickly process large volumes of information and generate contextually relevant responses suggests a role as supportive tools in both clinical and academic practice. At the same time, important concerns remain regarding their accuracy, consistency, and alignment with established scientific society guidelines, issues that are particularly relevant in highly complex fields such as GO.

Currently, the use of ChatGPT within the GO community remains unclear. In our cross-sectional study, we aimed to assess current practices and perceptions of ChatGPT among GO professionals.

## Methods

We conducted a cross-sectional study using an online survey developed and distributed via an online survey software, SurveyMonkey (https://www.surveymonkey.com/r/J3RLP2S). The questionnaire was disseminated through multiple social media channels (such as X, Instagram, LinkedIn, Facebook, and WhatsApp), supplemented by a snowball sampling approach and distribution via scientific social media networks. The study population targeted healthcare professionals involved in GO care across the globe, which includes Gynecologic Oncology Specialist, Gynecologic Oncology Fellow (In Training), and gynecologists. Participation was voluntary, and responses were collected anonymously. Data collection was conducted between July and September 2025, after which the survey link was deactivated.

We designed a 23-item multiple-choice survey, and the questions were independently reviewed by two senior gynecologic oncologists who were not part of the research team (Supplementary 1). In preparing the survey, we reviewed a previous validation study on assessing attitudes and usage of ChatGPT and subsequently finalized the survey for the gynecologic oncology community [9]. Demographic information collected included age, gender, type of institution, country of practice, years in practice, professional status, and academic title. Participants were asked a series of structured questions regarding their awareness, use, and perceptions of ChatGPT in GO. Items included prior awareness of ChatGPT, frequency and type of use (e.g., academic writing, literature summarization, patient communication, clinical decision support, teaching, and exam preparation), as well as specific areas of application within GO (e.g., endometrial, cervical, or ovarian cancer; sentinel lymph node mapping; adjuvant treatment planning; and risk stratification). Respondents were further asked about their use of ChatGPT in patient communication, tumor board preparation, and clinical decision-making. Perceptions of ChatGPT’s utility, reliability, contribution to professional work, and potential role in education and knowledge equalization were assessed using 5-point Likert scales (Strongly agree, Agree, Neutral, Disagree, Strongly disagree). Additional questions explored the version of ChatGPT used, perceived benefits, and concerns related to misinformation, data privacy, overreliance, and ethical/legal issues. The participants were asked whether they would recommend ChatGPT to colleagues, whether they believed artificial intelligence (AI) tools would become a standard component of clinical decision-making in the future, and to rate the perceived effectiveness of ChatGPT for academic writing, literature summarization, patient communication, clinical decision support, teaching, and exam preparation. The survey was administered in both Turkish and English, with the two languages presented concurrently throughout all questions. The forward–backward translation method was used for the translation of the questionnaire from English into Turkish. After translation, experts/experienced academicians evaluated the version to determine whether there was any loss of meaning. The translation was further reviewed by bilingual experts, and it was confirmed that semantic integrity was preserved. Our study was approved by the Ethics Committee of Başakşehir Çam and Sakura City Hospital under the approval number 259, dated August 27, 2025.

### Statistical analysis

Data were entered and analyzed using the Statistical Package for the Social Sciences (SPSS) Version 28.0 for Mac OS X (Chicago, IL, USA). Descriptive statistics were reported as frequencies and percentiles. Figures were generated using Microsoft^®^ Excel for Mac, Version 16.95.4. Categorical variables were analyzed using the Chi-squared test to assess associations between groups. A p-value of < 0.05 was considered statistically significant. In case of missing data, we accepted maximum of 5% based on the literature [10]. Descriptive statistics were summarized using frequencies, medians, and percentiles. Normality of the data was assessed with the Kolmogorov–Smirnov and Shapiro–Wilk tests, considering a distribution normal when the alpha level exceeded 0.05. Continuous variables with a normal distribution are reported as mean ± standard deviation (SD), while non-normally distributed variables are presented as medians with interquartile ranges (IQR; 25th–75th percentile). We analyzed the five-point likert question as follows for descriptive analysis: strongly agree = 5, agree = 4, neutral = 3, disagree = 2, and strongly disagree = 1.

## Results

During the data collection period, a total of 117 responses were obtained. After a careful assessment for completeness, 111 responses were used eligible for final analysis, remaining within the accepted threshold of a maximum 5% missing data. Therefore, six respondents were excluded due to incomplete responses.

We summarized the demographic and professional characteristics in Table 1. More than half of the participants were between 36 and 45 years of age(*n* = 61, 55.0%). Slightly over half identified as female(50.5%), while 44.1% identified as male, yielding a female-to-male ratio of 1.15:1. The majority of respondents were affiliated with academic institutions(*n* = 97, 87.4%), most commonly research and training hospitals(*n* = 58, 52.3%) and university hospitals(*n* = 39, 35.1%). Although participants represented eight different countries, the survey was predominantly completed by respondents from Türkiye(*n* = 100, 90.1%). More than one-third of participants reported less than five years of professional experience(*n* = 43, 38.7%), whereas nearly one in ten had over 20 years of experience. Over half of the participants(*n* = 72, 64.9%) were GO specialists. More than half of the participants(58.6%) did not hold an academic title, while 41.4% reported a faculty appointment ranging from assistant to full professor.

**Table 1:** Demographics of the participants

Variable	Number (*n* = 111, %)	Experience (< 5 years) (*n* = 43, %)	Experience (≥ 5 years) (*n* = 68, %)	*p*-value
Age (years)	*	†	‡	
- 25–35	24 (21.6)	21 (51.2)	3 (4.7)	**< 0.001**
- 36–45	61 (55.0)	19 (46.3)	42 (65.6)	
- 46–55	14 (12.6)	1 (2.3%)	13 (20.3)	
- 56–65	2 (1.8)	0 (0.0)	2 (3.1)	
- 66–75	4 (3.6)	0 (0.0)	4 (6.3)	
- > 75	0 (0.0)	0 (0.0)	0 (0.0)	
Gender				
- Female	56 (50.5)	25 (58.1)	31 (45.6)	0.436
- Male	49 (44.1)	16 (37.1)	33 (48.5)	
- Prefer not to specfy	6 (5.4)	2 (4.7)	4 (5.9)	
Type of institution				
- University hospital	39 (35.1)	12 (27.9)	27 (39.7)	**0.049**
- Research and Training hospital - City hospital	58 (52.3)	26 (60.5)	32 (47.1)	
- State hospital	7 (6.3)	5 (11.6)	2 (2.9)	
- Private hospital	5 (4.5)	0 (0.0)	5 (7.4)	
- Other	2 (1.8)	0 (0.0)	2 (2.9)	
Country of practice				
- Austria	1 (0.9)	1 (2.3)	0 (0.0)	0.370
- Georgia	1 (0.9)	0 (0.0)	1 (1.5)	
- Germany	4 (3.6)	3 (7.0)	1 (1.5)	
- India	2 (1.8)	0 (0.0)	2 (2.9)	
- Switzerland	1 (0.9)	0 (0.0)	1 (1.5)	
- Taiwan	1 (0.9)	0 (0.0)	1 (1.5)	
- Türkiye	100 (90.1)	39 (90.7)	61 (89.7)	
- Ukrain	1 (0.9)	0 (0.0)	1 (1.5)	
Years in practice				
- < 5 years	43 (38.7)	NA	NA	
- 5–10 years	30 (27.0)			
- 11–20 years	27 (24.3)			
- > 20 years	11 (9.9)			
Professional status				
- Gynecologic Oncology Specialist	72 (64.9)	18 (41.9)	54 (79.4)	**< 0.001**
- Gynecologic Oncology Fellow (In Training)	31 (27.9)	18 (41.9)	13 (19.1)	
- Gynecologists	8 (7.2)	7 (16.3)	1 (1.5)	
Academic title				
- None	65 (58.6)	39 (90.7)	26 (38.2)	**< 0.001**
- Assistant Professor	9 (8.1)	2 (4.7)	7 (10.3)	
- Associate Professor	26 (23.4)	1 (2.3)	25 (36.8)	
- Full Professor	11 (9.9)	1 (2.3)	10 (14.7)	

* Number of missing data = 6 (5.4%)

†Number of missing data = 2 (4.6%)

‡ Number of missing data = 4 (5.9%)

*NA* not applicable

Table 2 demonstrates the survey items on awareness, utilization, and perceptions of ChatGPT in clinical and academic practice. Nearly all the respondents(99.1%) had heard of ChatGPT. Two-thirds(*n* = 74, 66.7%) reported using it in their professional activities, most commonly on a weekly(*n* = 35, 31.5%) or daily(*n* = 32, 28.8%) basis. The free version(GPT-3.5) was used most frequently(45.0%), followed by the paid GPT-4o version(36.9%). ChatGPT was primarily used for literature summarization(*n* = 58, 52.3%), teaching(*n* = 47, 42.3%), and academic writing(*n* = 41, 36.9%), with additional use in exam preparation(*n* = 18, 16.2%), clinical decision support(*n* = 28, 25.2%), and minimally for patient communication(*n* = 8, 7.2%). In GO, most respondents reported using ChatGPT(*n* = 91, 82.0%), with the highest use in endometrial cancer (*n* = 62, 55.9%), followed by cervical cancer(*n* = 46, 41.4%). However, the direct clinical use remained limited, with only 9.0%(*n* = 10) using ChatGPT to explain diagnoses or treatments to patients and 17.1%(*n* = 19) for tumor board preparation or clinical decision-making. The primary concern identified was misinformation, reported by over two-thirds of respondents(*n* = 74), whereas only 5.4% indicated that they perceived no concerns regarding the use of ChatGPT in clinical practice. Despite these limitations, more than half of respondents(53.2%) believed that AI tools like ChatGPT will become a standard component of clinical decision-making.

**Table 2:** Questions on awareness, utilization, and perceptions of ChatGPT in clinical and academic practice

Variable	Number (*n*, %)	Experience (< 5 years) (*n* = 43, %)	Experience (≥ 5 years) (*n* = 68, %)	*p*-value
Heard of ChatGPT				
- Yes	110 (99.1)	42 (97.7)	68 (100.0)	0.207
- No	1 (0.9)	1 (2.3)	0 (0.0)	
Currently use ChatGPT in the professional activities	*	‡	§	
- Yes	74 (66.7)	35 (85.4)	39 (60.9)	**0.007**
- No	31 (27.9)	6 (14.6)	25 (39.1)	
How often do you use ChatGPT?				
- Daily	32 (28.8)	18 (41.9)	14 (20.6)	**0.021**
- Weekly	35 (31.5)	16 (37.2)	19 (27.9)	
- Monthly	9 (8.1)	1 (2.3)	8 (11.8)	
- Rarely	20 (18.0)	5 (11.6)	15 (22.1)	
- Never	15 (13.5)	3 (7.0)	12 (17.6)	
Which version of ChatGPT do you primarily use?				
- Free version (GPT-3.5)	50 (45.0)	19 (44.2)	31 (45.6)	0.415
- Paid version (GPT-4o)	41 (36.9)	19 (44.2)	22 (32,4)	
- don’t know	6 (5.4)	2 (4.7)	4 (5.9)	
- do not use it	14 (12.6)	3 (7.0)	11 (16.2)	
For what purposes do you use ChatGPT? †				
- Academic writing	41 (36.9)	21 (48.8)	20 (29.4)	**0.039**
- Literature summary	58 (52.3)	27 (62.8)	31 (45.6)	0.077
- Patient communication	8 (7.2)	4 (9.3)	4 (5.9)	0.497
- Clinical decision support	28 (25.2)	11 (25.6)	17 (25.0)	0.945
- Teaching	47 (42.3)	22 (51.2)	25 (36.8)	0.135
- Exam preparation	18 (16.2)	9 (20.9)	9 (13.2)	0.284
- never used	17 (15.3)	4 (9.3)	13 (19.1)	0.162
In which gynecologic oncology areas have you used ChatGPT? †				
- Endometrial cancer	62 (55.9)	27 (62.8)	35 (51.5)	0.242
- Cervical cancer	46 (41.4)	21 (48.8)	25 (36.8)	0.208
- Ovarian cancer	42 (37.8)	20 (46.5)	22 (32.4)	0.134
- SLN mapping or surgical guidelines	35 (31.5)	17 (39.5)	18 (26.5)	0.149
- Adjuvant treatment planning	30 (27.0)	14 (32.6)	16 (23.5)	0.297
- Risk stratification or staging	39 (35.1)	18 (41.9)	21 (30.9)	0.238
- I Never Use It	20 (18.0)	7 (16.3)	13 (19.1)	0.705
Have you ever used ChatGPT to help explain a cancer diagnosis or treatment to a patient?		7 (16.3)	3 (4.4)	
- Yes	10 (9.0)	36 (83.7)	65 (95.6)	**0.033**
- No	101 (91.0)			
Have you ever used ChatGPT to help prepare for a tumor board discussion or clinical decision making?	*	‡	§	
- Yes	19 (17.1)	10 (24.4)	9 (14.1)	0.180
- No	86 (77.5)	31 (75.6)	55 (85.9)	
Have you encountered inaccurate or misleading medical content from ChatGPT?				
- Yes	59 (53.2)	29 (67.4)	30 (44.1)	0.056
- No	34 (30.6)	9 (20.9)	25 (36.8)	
- Never Use It	18 (16.2)	5 (11.6)	13 (19.1)	
What are your main concerns with ChatGPT in clinical use?	†			
- Misinformation	74 (66.7)	32 (74.4)	42 (61.8)	0.168
- Data privacy	57 (51.4)	26 (60.5)	31 (45.6)	0.127
- Overreliance	36 (32.4)	12 (27.9)	24 (35.3)	0.418
- Ethical-legal issues	37 (33.3)	13 (30.2)	24 (35.3)	0.582
- None	6 (5.4)	1 (2.3)	5 (7.4)	0.254
- Never Use It	14 (12.6)	6 (14.0)	8 (11.8)	0.735
Do you think AI tools like ChatGPT will become a standard part of clinical decision-making in the future?	*	‡	§	
- Yes	59 (53.2)	27 (65.9)	32 (50.0)	0.150
- No	3 (2.7)	0 (0.0)	3 (4.7)	
- Maybe	43 (38.7)	14 (32.6)	29 (45.3)	

* Number of missing data = 6 (5.4%)

† Multiple responses were allowed

‡ Number of missing data = 2 (4.6%)

§ Number of missing data = 4 (5.9%)

In our survey, 53.2% of respondents reported encountering inaccurate or misleading medical content generated by ChatGPT. This was more commonly reported by participants with less than 5 years of experience (67.4%) compared with those with ≥ 5 years of experience (44.1%), although the difference did not reach statistical significance (p-value = 0.056).

**Fig. 1:**
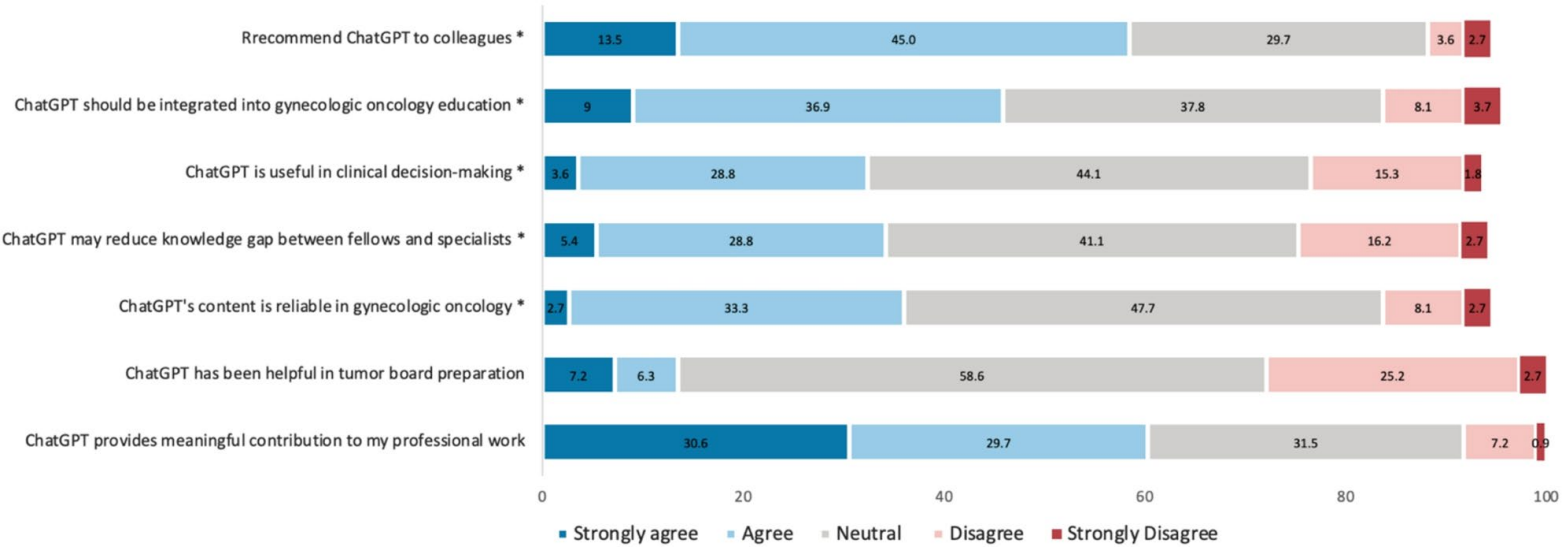
Agreement levels with statements regarding ChatGPT’s role in clinical practice and education. * Number of missing data = 6 (5.4%)

The distribution of responses to the 5-point Likert scale questions is presented in Fig. 1. Overall, 60.3% of participants agreed or strongly agreed that ChatGPT makes a meaningful contribution to their professional work, while 8.1% disagreed or strongly disagreed (median 4, IQR:3–5). In contrast, when asked about its usefulness in tumor board preparation, the majority(58.6%) remained neutral, with only 13.5% expressing agreement and 27.9% disagreement (median 3, IQR:2–3). Regarding reliability in GO, most respondents reported a neutral stance(47.7%), whereas 36.0% considered the content reliable and 10.8% found it unreliable (median 3, IQR:3–4). Similarly, 34.2% of participants agreed that ChatGPT may help bridge the knowledge gap between fellows and specialists, although 41.4% remained neutral and 18.9% disagreed (median 3, IQR:3–4). Perceptions of ChatGPT’s role in clinical decision-making reflected a similar trend, with 31.4% agreement, 44.1% neutrality, and 17.1% disagreement (median 3, IQR:3–4). On the topic of integration into GO education, nearly half(45.9%) endorsed its inclusion, 37.8% were neutral, and 11.8% opposed it (median 3, IQR:3–4). Moreover, 58.5% of respondents reported they would recommend ChatGPT to colleagues, while only 6.3% expressed disagreement (median 4, IQR:3–4).

In comparisons between ChatGPT users and non-users, users demonstrated significantly stronger agreement in its recommendation than non-users (p-value = < 0.001). As expected, non-users were more likely to disagree with the integration of ChatGPT into gynecologic oncology practice (p-value = 0.003). Similarly, non-users expressed greater disagreement regarding the reliability of ChatGPT in gynecologic oncology (p-value = < 0.001). The users showed significantly higher agreement than non-users regarding the use of ChatGPT in tumor board preparation (p-value = < 0.001). However, there was no statistically significant difference between the groups in their belief that ChatGPT may reduce the gap between specialists and fellows (p-value = 0.166).

**Fig. 2:**
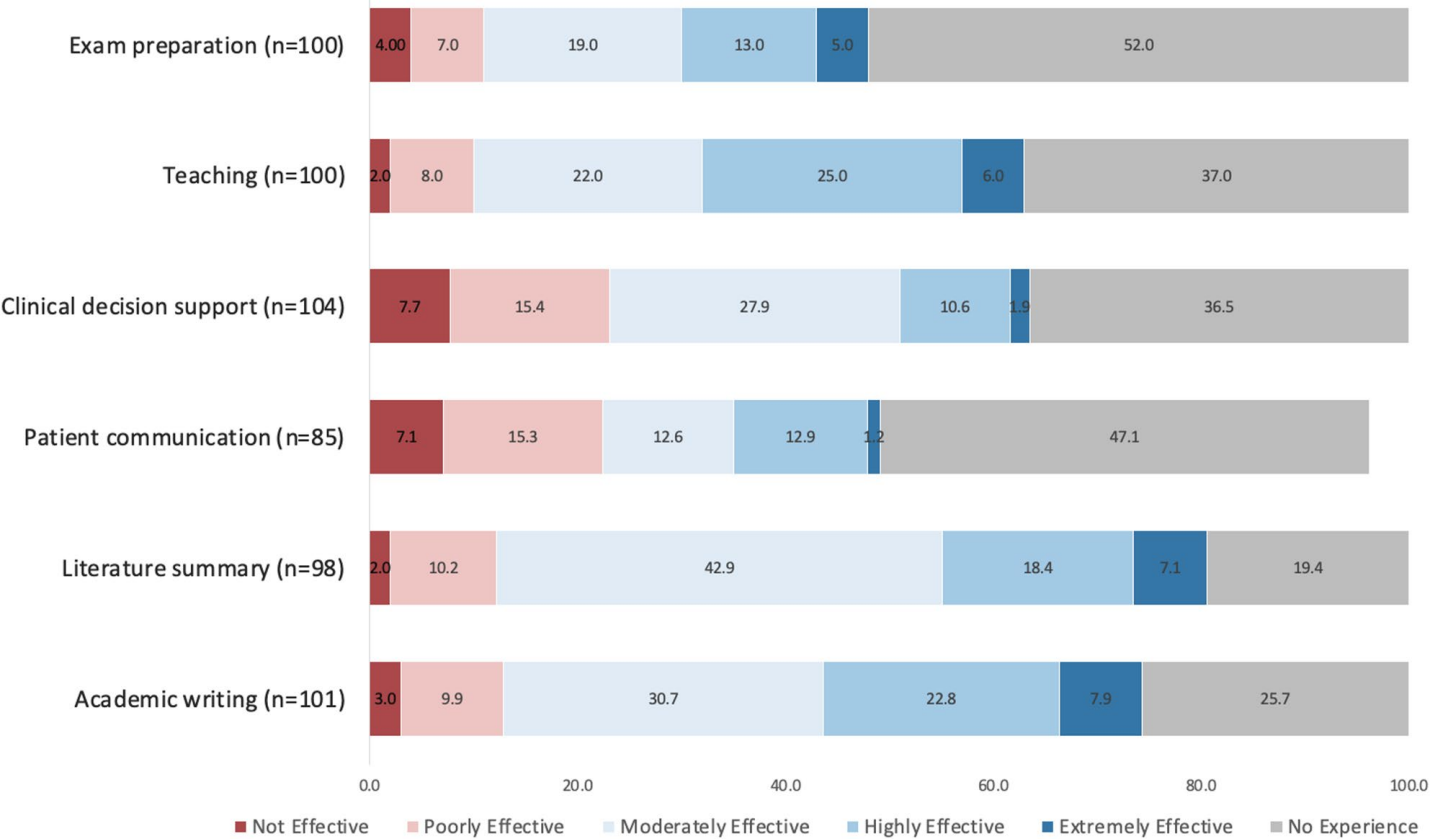
Distribution of perceived effectiveness of ChatGPT in writing, teaching, clinical support, and communication

Figure 2 illustrates the distribution of participants’ perceptions regarding the effectiveness of ChatGPT across different domains, including academic writing, literature summarization, patient communication, clinical decision support, teaching, and exam preparation. Herein, we reported only the fully completed responses, and the corresponding total numbers for each category are presented in the figure. In academic writing, 30.7% rated ChatGPT as moderately effective, while 22.8% considered it highly effective; however, over a quarter reported no prior experience. A similar pattern was observed for literature summarization, with 42.9% rating it moderately effective and 18.4% highly effective, though 19.4% had no experience. In contrast, perceptions were less favorable for patient communication, where 47.1% reported no experience and only 14.1% rated ChatGPT as highly or extremely effective. For clinical decision support, 27.9% perceived moderate effectiveness, while 36.5% had no prior experience. Teaching showed more balanced opinions, with 25.0% rating ChatGPT as highly effective and 22.0% as moderately effective, although 37.0% had not used it. Furthermore, exam preparation demonstrated the lowest uptake, with over half of participants(52.0%) reporting no experience and relatively few finding it highly(13.0%) or extremely(5.0%) effective.

Further comparative analyses revealed that participants older than 45 years used ChatGPT in their professional activities significantly less often than those younger than 45 years(p-value = 0.026). Similarly, the frequency of ChatGPT use was higher among participants younger than 45 years compared with those older than 45 years(p-value = 0.015). However, there was no significant difference in the use of the paid version of ChatGPT between these two age groups(p-value = 0.214). For clinical decision-making, participants younger than 45 years reported significantly more frequent use compared with those older than 45 years(p-value = 0.013).

Participants with less than 5 years of clinical experience reported using ChatGPT in their professional activities significantly more often than those with more than 5 years of experience(p-value = 0.007). Similarly, ChatGPT use was more frequent among those with less than 5 years of experience compared to their more experienced counterparts(p-value = 0.021). In contrast, participants with more than 5 years of clinical experience reported using ChatGPT more frequently for writing purposes compared with those with less than 5 years of experience(p-value = 0.039).

## Discussion

Our cross-sectional survey provides the snapshot of how GO professionals perceive and use ChatGPT. It shows broad awareness but varied adoption with strong interest in academic and educational use, yet cautious integration into clinical practice. Our participants (*n* = 111) were predominantly aged 36–45 years, affiliated with academic institutions, and mostly based in Türkiye, with over half being gynecologic oncologists, and slightly more female than male. Nearly all respondents were aware of ChatGPT, and two-thirds reported using it professionally, primarily for literature summarization (52.3%), teaching (42.3%), and academic writing (36.9%), whereas direct clinical use remained limited (≤ 17.1%). Misinformation and data privacy were the most commonly reported concerns, yet over half anticipated AI tools like ChatGPT becoming standard in clinical decision-making in the future. Perceptions of effectiveness varied by domain: ChatGPT was viewed as moderately or highly effective in academic tasks(writing, summarization, teaching) but less so for patient communication, clinical decision support, or exam preparation. Overall, over half of the respondents believed ChatGPT meaningfully contributed to their professional work, nearly half supported its integration into GO education, and 58.5% indicated they would recommend it to colleagues. Participants younger than 45 years and those with less than 5 years of clinical experience reported significantly more frequent use of ChatGPT in their professional activities and for clinical decision-making (p-value = 0.026 and 0.015, respectively). In contrast, participants with greater clinical experience were more likely to use ChatGPT for writing purposes (p-value = 0.039), with no difference observed in the use of the paid version across age groups.

Consistent with a previous systematic review, ChatGPT is primarily used for text-based tasks, as also observed in our study [1]. In the literature, misinformation remains a leading concern, particularly in amplifying existing misinformation in healthcare-related topics, such as about vaccines in large language models [11, 12]. Similarly, our study identified misinformation as the primary concern among respondents.

Although a previous study reported that ChatGPT provided higher-quality recommendations for ovarian cancer compared to endometrial cancer, our study found that it was most frequently used for information related to endometrial cancer [5]. One possible explanation might due to its higher prevalence among gynecologic malignancies [13]. Furthermore, another possible explanation is that the management of endometrial cancer has become increasingly complex with the introduction of molecular classification, which requires more detailed pathological and genetic testing. As a result, guideline recommendations from different scientific societies, such as ESGO, NCCN, and various national guidelines, have grown more complex and sometimes divergent [5]. This complexity further challenges ChatGPT’s performance in endometrial cancer, making it more difficult to accurately apply and recall recommendations during the clinical decision-making process.

The systematic review of LLMs as consultation platforms or clinical decision support systems in gynecologic oncology concluded that there is considerable variability in how studies address interaction bias and implement benchmarking approaches [5]. Consistent with this, in our study, participants’ main concern regarding ChatGPT in clinical use, misinformation (66.7%) reflects this underlying variability and potential for bias.

A cross-sectional study among GO fellows in the USA reported ChatGPT use at 53.1%, which is comparable to 66.7% in our study [14]. Moreover, Sciuva et al. reported ChatGPT use for academic writing purposes at 35.3%, which was similar to the rate observed in our study(36.9%) [14]. Regarding the use of ChatGPT in direct patient care, 11.8% of respondents agreed in the study by Sciuva et al., compared with 28.8% in our study, where a similar item assessed the perceived usefulness of ChatGPT in clinical decision-making [14]. Such differences may reflect variations in physicians’ training backgrounds, healthcare system structures, or geographical settings. In comparison to this US-based paper (*n* = 36), our study includes a larger, multicountry cohort (*n* = 111) and uniquely reports on task-specific effectiveness across academic, educational, and clinical domains.

In a systematic review of 65 studies on LLMs in healthcare, ChatGPT was found to be effective in summarizing medical documents for a range of purposes, including the synthesis of research articles [1]. This aligns with our findings, where 52.3% of the participants reported using ChatGPT for literature summarization. ChatGPT has shown potential for achieving high accuracy in diagnosing certain diseases [1]; however, to date, no studies or meta-analyses have specifically evaluated its performance in gynecologic oncology. In addition, ChatGPT can suggest guideline-based treatment strategies for advanced solid tumors, such as breast cancer, and has been applied to predict and explain potential drug–drug interactions [1]. Despite these promising capabilities, most studies have highlighted ongoing technical and ethical challenges associated with its use [1].

Another cross-sectional study evaluating surgeons’ awareness, expectations, and involvement with ChatGPT reported that an overwhelming 96.6% of respondents expressed a willingness to integrate AI into their clinical practice [15]. Interestingly, this far exceeds the proportion of participants in our study who reported having used ChatGPT to assist with tumor board preparation or clinical decision-making, which was only 17.1%. Additionally, the prevalence of ethical concerns was markedly lower in our cohort (33.3%) compared to the 87.2% reported by Arboit et al. [15]. A survey of 200 participants on the use of artificial intelligence in emergency and trauma surgery reported that 61.5% considered AI useful for training and education [16]. In our study, a similar question regarding ChatGPT’s integration into gynecologic oncology education found that 45.9% of participants supported its inclusion.

A survey of 650 surgeons from 71 countries across five continents in the trauma and emergency surgions concluded that there is a split between them who are confident in AI’s potential and those who are skeptical or hesitant to trust AI-based surgical decision-support tools [17]. Similarly, in our survey, 53.3% of participants responded “yes” to the question of whether AI tools like ChatGPT will become a standard part of clinical decision-making in the future, reflecting a comparable split.

Evidently, a major limitation is the absence of a pre-determined sample size, since the survey was distributed online and internationally, which could impact the representativeness and generalizability of our findings. Due to the exploratory nature of this study and the relatively limited sample size, we did not perform multivariable regression analyses to assess associations between ChatGPT use and demographic or professional variables. Moreover, lack of report on the response rate was due to use of multiple online recruitment channels. Additionally, the survey questions were not standardized, and no validated scoring systems specific to the topic were used, potentially introducing variability in responses. The study focused solely on a single large language model, ChatGPT, which limits the assessment of other large language models. Although the survey included participants from eight countries, the majority were from Türkiye, further constraining generalizability. While we assessed ChatGPT’s use across different gynecologic cancers and clinical settings, we did not evaluate the accuracy, completeness, or reliability of the information provided. For example, although endometrial cancer was the condition most frequently queried, it remains unclear how participants assessed the quality or usefulness of the information obtained. Currently, the literature has primarily focused on the use of LLMs in general; however, their adoption and utilization by physicians within the GO community have not been systematically evaluated. Despite the aforementioned limitations, our cross-sectional study provides valuable insights into physicians’ perspectives and actual use of LLMs in clinical practice. The strength of our study included the large and diverse sample of GO professionals, the balance between academic and clinical users, and the timely assessment of ChatGPT use soon after its release.

Future studies with larger and more diverse cohorts are warranted to explore relationships between ChatGPT use and demographic or professional variables in greater depth and to provide more robust, generalizable insights. Future research in GO should focus on optimizing LLMs for patient-facing applications, evaluating their integration into multidisciplinary tumor boards, and assessing potential ethical, legal, and safety considerations in clinical practice. Additionally, longitudinal studies are warranted to measure how continuous exposure to and training with LLMs affects clinician workflow efficiency, patient outcomes, and the quality of medical education. Future studies should evaluate and compare the outcomes of diverse LLMs to explore their use by the GO community.

## Conclusions

Our findings suggest that while ChatGPT demonstrates moderate to high perceived effectiveness in academic writing, literature summarization, and teaching, its utility in patient communication and clinical decision support remains limited. This indicates that LLMs may serve as valuable adjuncts for scholarly and educational tasks but should be used cautiously in direct clinical interactions until their accuracy, empathy, and completeness are further validated. Taken together, concerns regarding information security and ethical considerations emphasize that, despite enthusiasm for AI integration, its successful implementation in clinical practice requires careful attention to technical, ethical, and legal aspects to ensure patient safety and maintain trust.

## Supplementary Information

Supplementary Material 1.

## Data Availability

The data is available upon request.

## Abbreviations

LLM: Large language models
ChatGPT: Chat Generative Pre-Trained Transformer
GO: Gynecologic oncology
NCCN®: National Comprehensive Cancer Network^®^
ESGO: European Society of Gynaecological Oncology
ChatGPT-4o: Chat Generative Pre-Trained Transformer–omni
AI: Artificial intelligence
SPSS: Statistical Package for the Social Sciences
