# Supplementary material for: Current practices and perceptions of ChatGPT in gynecologic oncology: results from a cross-sectional questionnaire (TRSGO-AI-001)
Source: BMC Med Educ. 2026 Apr 15;26:617. doi: 10.1186/s12909-026-09192-w (PMC13081464; doi:10.1186/s12909-026-09192-w)
Supplement: Supplementary file 1 — Supplementary Material 1. [file 12909_2026_9192_MOESM1_ESM.pdf]

Değerli Meslektaşlarım,

Jinekolojik onkoloji uzmanları ve uzmanları arasında ChatGPT ve diğer üretken yapay zeka araçlarının farkındalığını, kullanım modellerini ve algılanan faydalarını veya sınırlamalarını araştırmayı amaçlayan kısa bir ankete katılmanızı rica ediyoruz. Yapay zeka tıbbın geleceğini şekillendirmeye devam ederken, katkılarınız bu teknolojilerin alanımızdaki klinik uygulama, araştırma ve eğitime nasıl entegre edildiğine dair değerli bilgiler sağlayacaktır. Anketin tamamlanması yaklaşık 3-5 dakika sürecek ve tüm yanıtlar anonim kalacak ve yalnızca toplu olarak raporlanacaktır.

Dear Colleagues,
We kindly invite you to participate in a brief survey aimed at exploring the awareness, usage patterns, and perceived benefits or limitations of ChatGPT and other generative AI tools among gynecologic oncology specialists and fellows. As artificial intelligence continues to shape the future of medicine, your input will provide valuable insights into how these technologies are currently integrated into clinical practice, research, and education within our field. The survey will take approximately 3-5 minutes to complete, and all responses will remain anonymous and be reported only in aggregate form.

By continuing with the survey, I confirm that I have read the information provided and give my informed consent to participate.

* 1. Demographics / Demografik Bilgiler
Country of practice / Çalışma ülkeniz:

- ◯ Türkiye
- ◯ Other /Diğer (Please write).........................

[ ]

* 2. Your gender/Cinsiyetiniz

- ◯ Female/Kadın
- ◯ Male/Erkek
- ◯ I don't want to specify/Belirtmek istemiyorum

3. What is your current age range?/Şu anki yaş aralığınız kaç ?

- ◯ 25-35
- ◯ 36-45
- ◯ 46-55
- ◯ 56-65
- ◯ 66-75
- ◯ >75

* 4. Type of institution / Kurum tipi:

- ◯ University hospital / Üniversite hastanesi
- ◯ Research and Training hospital - City hospital / Eğitim araştırma hastanesi -Şehir Hastanesi
- ◯ State hospital / Devlet hastanesi
- ◯ Private hospital / Özel hastane
- ◯ Other (please specify)./Diğer (lütfen belirtiniz)......

* 5. Years in practice / Uzmanlık süreniz:

- ◯ <5 years / 5 yıldan az
- ◯ 5–10 years
- ◯ 11–20 years
- ◯ >20 years / 20 yıldan fazla

* 6. Your professional status / Mesleki statünüz:

- ◯ Gynecologic Oncology Specialist / Jinekolojik Onkoloji Uzmanı
- ◯ Gynecologic Oncology Fellow (In Training) / Jinekolojik Onkoloji Yan Dal Asistanı
- ◯ Other (please specify)/Diğer (lütfen belirtiniz).......

* 7. Academic title / Akademik unvanınız:

- ◯ None / Yok
- ◯ Assistant Professor / Dr. Öğr. Üyesi
- ◯ Associate Professor / Doçent
- ◯ Professor / Profesör

* 8. Awareness & Access / Farkındalık ve Erişim
Have you heard of ChatGPT? / ChatGPT'yi duydunuz mu?

- ◯ Yes / Evet
- ◯ No / Hayır

* 9. Do you currently use ChatGPT in your professional activities ?/Mesleki faaliyetlerinizde ChatGPT kullanıyor musunuz?

- ◯ Yes/Evet
- ◯ No/Hayır

* 10. ChatGPT provides meaningful contribution to my professional work /ChatGPT profesyonel çalışmalarıma anlamlı katkı sağlıyor

- ◯ Strongly agree/Kesinlikle katılıyorum
- ◯ Agree/Katılıyorum
- ◯ Neutral /Kararsızım
- ◯ Disagree/Katılmıyorum
- ◯ Strongly disagree/Kesinlikle katılmıyorum

* 11. How often do you use ChatGPT? / ChatGPT'yi ne sıklıkla kullanıyorsunuz?

- ◯ Daily / Her gün
- ◯ Weekly / Haftalık
- ◯ Monthly / Aylık
- ◯ Rarely / Nadiren
- ◯ Never / Hiç kullanmadım

* 12. Which version of ChatGPT do you primarily use? Please choose one / ChatGPT'nin hangi sürümünü ağırlıklı olarak kullanıyorsunuz? lütfen birini seçiniz

- ◯ Free version (GPT-3.5) / Ücretsiz sürüm (GPT-3.5)
- ◯ Paid version (GPT-4o) / Ücretli sürüm (GPT-4o)
- ◯ I don't know / Bilmiyorum
- ◯ I do not use ChatGPT / ChatGPT kullanmıyorum

* 13. Usage Behavior / Kullanım Alışkanlıkları
For what purposes do you use ChatGPT? (Select all that apply) / ChatGPT'yi hangi amaçlarla kullanıyorsunuz? (Birden fazla seçilebilir)

- ☐ Academic writing / Akademik yazım
- ☐ Literature summary / Literatür özeti
- ☐ Patient communication / Hasta bilgilendirme
- ☐ Clinical decision support / Klinik karar desteği
- ☐ Teaching / Eğitim materyali
- ☐ Exam preparation / Sınav hazırlığı
- ☐ Hiç kullanmıyorum/I Never Use It....

[ ]

* 14. How effective do you personally find ChatGPT for the following purpose?, Please rate the usefulness of ChatGPT /Aşağıdaki amaçlar için ChatGPT'yi kişisel olarak ne kadar etkili buluyorsunuz? Lütfen ChatGPT'nin yararlılığını değerlendirin

	Not effective/Etkili Değil	Poorly effective/Zayıf etkili	Moderately effective /Orta etkili	Highly effective /Yüksek etkili	Extremely effective / Son derece etkili	No experience/Deneyimim yok
Academic writing /Akademik yazım	◯	◯	◯	◯	◯	◯
Literature summary /Literatür özeti	◯	◯	◯	◯	◯	◯
Patient Communication /Hasta bilgilendirme	◯	◯	◯	◯	◯	◯
Clinical desicion support /Klinik Karar desteği	◯	◯	◯	◯	◯	◯
Teaching /Eğitim materyali	◯	◯	◯	◯	◯	◯
Exam preparation/Sınav hazırlığı	◯	◯	◯	◯	◯	◯

* 15. In which gynecologic oncology areas have you used ChatGPT? (Multiple answers possible) / ChatGPT’yi jinekolojik onkolojide hangi konularda kullandınız? (Çoklu seçim yapılabilir)

- ☐ Endometrial cancer/Endometrium kanseri
- ☐ Cervical cancer/ Serviks kanseri
- ☐ Ovarian cancer/Over kanseri
- ☐ SLN mapping or surgical guidelines/SLN haritalaması veya cerrahi kılavuzlar
- ☐ Adjuvant treatment planning/Adjuvan tedavi planlaması
- ☐ Risk stratification or staging/Risk sınıflandırması veya evrelemesi
- ☐ Hiç kullanmıyorum/I Never Use It....

* 16. Have you ever used ChatGPT to help explain a cancer diagnosis or treatment to a patient? / ChatGPT’yi bir hastaya tanı veya tedavi sürecini açıklamak için kullandınız mı?

- ◯ Yes / Evet
- ◯ No / Hayır

* 17. Have you ever used ChatGPT to help prepare for a tumor board discussion or clinical desicion making ?/ChatGPT'yi tümör konseyine hazırlık veya klinik karar desteği için kullandınız mı ?

- ◯ Yes /Evet
- ◯ No /Hayır

* 18. ChatGPT has been helpful in tumor board preparation/ChatGPT tümör konseyine hazırlıkta faydalı olmaktadır

- ◯ Strongly agree/Kesinlikle katılıyorum
- ◯ Agree/Katılıyorum
- ◯ Neutral /Kararsızım
- ◯ Disagree/Katılmıyorum
- ◯ Strongly disagree/Kesinlikle katılmıyorum

* 19. Please rate the following statements/Lütfen aşağıdaki ifadeleri değerlendirin

	1-Strongly disagree /Kesinlikle katılmıyorum	2-Disagree/Katılmıyorum	3-Neutral/ Kararsızım	4-Agree /Katılıyorum	5-Strongly agree /Kesinlikle katılıyorum
ChatGPT's content is reliable in gynecologic oncology/ChatGPT'nin içeriği jinekolojik onkolojide güvenilirdir	◯	◯	◯	◯	◯
ChatGPT may reduce knowlodge gap between fellows and specialists/ChatGPT, akademisyenler ve uzmanlar arasındaki bilgi farkını azaltabilir	◯	◯	◯	◯	◯
ChatGPT is useful in clinical desicion - making/ChatGPT klinik karar almada faydalıdır	◯	◯	◯	◯	◯
ChatGPT should be integrated into gynecologic oncology education/ChatGPT jinekolojik onkoloji eğitimine entegre edilmelidir	◯	◯	◯	◯	◯

* 20. Have you encountered inaccurate or misleading medical content from ChatGPT? / ChatGPT’den hatalı veya yanıltıcı tıbbi içerik aldınız mı?

- ◯ Yes / Evet
- ◯ No / Hayır
- ◯ Hiç kullanmadım /I Never Use It...

* 21. What are your main concerns with ChatGPT in clinical use? (Select all that apply) / Klinik kullanıma yönelik ChatGPT ile ilgili başlıca endişeleriniz nelerdir? (Çoklu seçim yapılabilir)

- ☐ Misinformation / Yanıltıcı bilgi
- ☐ Data privacy / Veri güvenliği
- ☐ Overreliance / Aşırı bağımlılık
- ☐ Ethical-legal issues / Etik-hukuki sorunlar
- ☐ None / Yok
- ☐ Hiç kullanmadım /I Never Use It

* 22. I would recommend ChatGPT to colleagues/Meslektaşlarıma ChatGPT'yi tavsiye ederim

- ☐ Strongly agree /Kesinlikle katılıyorum
- ☐ Agree /Katılıyorum
- ☐ Neutral /Kararsızım
- ☐ Disagree /Katılmıyorum
- ☐ Strongly disagree /Kesinlikle katılmıyorum

* 23. Do you think AI tools like ChatGPT will become a standard part of clinical decision-making in the future? / Sizce ChatGPT gibi yapay zekâ araçları gelecekte klinik kararların standart bir parçası olacak mı?

- ○ Yes /Evet
- ○ No /Hayır
- ○ Maybe /Belki
